# The Effect of White Light Spectrum Modifications by Excess of Blue Light on the Frost Tolerance, Lipid- and Hormone Composition of Barley in the Early Pre-Hardening Phase

**DOI:** 10.3390/plants12010040

**Published:** 2022-12-22

**Authors:** Mohamed Ahres, Tamás Pálmai, Terézia Kovács, László Kovács, Jozef Lacek, Radomira Vankova, Gábor Galiba, Péter Borbély

**Affiliations:** 1 Centre for Agricultural Research, Agricultural Institute, Eötvös Loránd Research Network, H-2462 Martonvásár, Hungary; 2 Biological Research Centre, Institute of Plant Biology, H-6701 Szeged, Hungary; 3 Institute of Experimental Botany of the Czech Academy of Sciences, 165 02 Prague, Czech Republic; 4 Department of Experimental Plant Biology, Faculty of Science, Charles University, 128 00 Prague, Czech Republic; 5 Department of Agronomy, GEORGIKON Campus, Hungarian University of Agricultural and Life Sciences, 8360 Keszthely, Hungary

**Keywords:** barley, cold acclimation, light-emitting diode (LED) lighting, phytohormones, lipidome, light regulation

## Abstract

It is well established that cold acclimation processes are highly influenced, apart from cold ambient temperatures, by light-dependent environmental factors. In this study we investigated whether an extra blue (B) light supplementation would be able to further improve the well-documented freezing tolerance enhancing effect of far-red (FR) enriched white (W) light. The impact of B and FR light supplementation to white light (WFRB) on hormone levels and lipid contents were determined in winter barley at moderate (15 °C) and low (5 °C) temperatures. Low R:FR ratio effectively induced frost tolerance in barley plantlets, but additional B light further enhanced frost hardiness at both temperatures. Supplementation of WFR (white light enriched with FR light) with B had a strong positive effect on abscisic acid accumulation while the suppression of salicylic acid and jasmonic acid levels were observed at low temperature which resembles the shade avoidance syndrome. We also observed clear lipidomic differences between the individual light and temperature treatments. WFRB light changed the total lipid content negatively, but monogalactosyldiacylglycerol (MGDG) content was increased, nonetheless. Our results prove that WFRB light can greatly influence phytohormone dynamics and lipid contents, which eventually leads to more efficient pre-hardening to avoid frost damage.

## 1. Introduction

Temperate, overwintering plants developed a unique capability to increase their freezing tolerance by the so-called cold acclimation at low but not freezing temperatures during late fall [1,2]. This genetically determined frost tolerance is achieved through a process of cold hardening, lasting for several weeks in cereals. It is well established that the cold acclimation process of winter-hardy plants is highly influenced, apart from cold ambient temperatures, by other light-dependent environmental factors like day length, the circadian clock, and the intensity and spectrum of the incident light [3,4,5]. C-repeat-binding factors (*CBF*s), as a transcription factor family plays an important role in cold acclimation both in monocot and dicot plant species. They up-regulate the expression of cold-responsive genes (CORs), whose expression results in a more pronounced freezing tolerance [6,7].

It was reported first in Arabidopsis and later also in winter varieties of wheat and barley that the freezing tolerance of plants in the early pre-hardening phase increased at non-acclimating temperatures (15 and 16 °C) when they were illuminated by white light (W) with low Red:Far-red (R:FR) ratios [8,9,10]. However, this light-quality-induced pre-hardening is much lower than what can be achieved by full cold hardening at temperatures around 0 °C.

Plants acclimate to freezing stress by increasing the degree of fatty acid unsaturation of membrane lipids and shortening hydrophobic fatty acyl chains to maintain membrane fluidity [11,12]. A balance between bilayer-forming galactolipids, such as digalactosyldiacyl glycerol (DGDG) and non-bilayer-forming monogalactosyl diacylglycerol (MGDG) together with a sufficient content of acidic sulfoquinovosyldiacylglycerol and phosphatidylglycerol (PG) in the chloroplast membranes is required to maintain membrane stability and activities of key proteins associated with photosynthesis [13]. Similarly, in extra-chloroplast membranes, proportions between the two most abundant classes of phosphoglycerolipids, bilayer-forming phosphatidylcholine (PC) and non-bilayer-forming phosphatidylethanolamine (PE) are crucial for their stability. In addition to temperature, light quality can also have an enormous effect on lipid composition. Previously, the ratios of MGDG/DGDG and PC/PE, increases in phosphatidic acid (PA) content [3,14], changes in the amount and composition of sphingolipids (SLs) [15,16,17,18,19,20] and diacylglycerol acyltransferase signaling [21] were identified as key elements in the response of plants to cold treatment. Application of white light enriched in far-red light (FR) resulted in elevated PG and phosphatidylserine (PS) levels in barley leaves at 15 °C compared to treatment with W [3]. At lower temperature (5 °C), FR supplementation increased the levels of several lipid classes, namely MGDG, PG, PE, phosphatidylinositol (PI) and PS already after 1 day. PE, PS, and PG are prominent lipid classes, which have important roles in signal transduction processes [22].

During the process of stress acclimation, phytohormones are very important. They play a key role in the regulation of plant interactions with the altering environment, since they are involved in the acclimatization processes [23,24]. In response to abiotic stresses, like drought, salinity or cold stress, ABA plays an essential role [25,26]. In the early phase of cold stress response, the elevation of ABA content can stabilize plant water homeostasis and induce the production of protective compounds to avoid cold injury [27,28]. SA is known to play an important role in stress responses of numerous plant species [29]. It has a positive effect on tolerance of low temperature in cereals [30,31]. In barley, exogenous SA application improved cold tolerance by decreasing lipid peroxidation as well as ice nucleation, regardless of the cold sensitivity of the varieties. The importance of JA is also crucial in many biotic and abiotic stress responses [32,33]. Transcription of JA biosynthetic genes and JA content are increased during cold stress, e.g., in rice [34]. Although auxins are a well-studied group of hormones, their role in cold acclimation is poorly understood: The accumulation of the biologically most active auxin, indole-3-acetic acid (IAA) as well as the expression of many genes involved in auxin metabolism are positively influenced by prolonged cold stress [35,36].

Beside temperature, the spectral composition of the light also affects plant cold acclimation [10,22]. We found recently that FR supplementation enhanced freezing tolerance at 15 °C, which is associated with increased levels of phytohormones like ABA, IAA and cis-zeatin. We also reported in our previous article that the combination of cold (5 °C) and FR enrichment further increased the level of ABA and was associated with a more pronounced increment in IAA and cis-zeatin content [5]. This phenomenon is mostly related to the well-known shade avoidance syndrome (SAS). SAS is initiated by the change from red (R) to far-red (FR) illumination and usually occurs in dense canopy [37]. According to the literature, the most prominent phenomena of SAS are elongation of internodes and increased stem lengths. This is accompanied by increased apical dominance, which leads to vertical growth to avoid shade [38]. Consequently, elevated proportions of FR light initiate the shade avoidance response via a number of molecular mechanisms, including up-regulation of auxins, gibberellins and brassinosteroids with down-regulation of JA and SA [39,40,41,42]. Side effects of SAS can also affect many other processes through changes in phytohormones.

In contrast to FR, until very recently, little evidence has accumulated about the possible involvement of blue light (B) in cold acclimation. It is important since as the solar zenith angle increases, the amount of blue light in the sunlight decreases relative to red light resulting in reddish sunsets and sunrises. In addition to that, the useful irradiance available at the surface is affected by many atmospheric factors such as aerosol and by the quantity and types of clouds [43,44]. The breakthrough happened in 2021, when several papers describing the possible role of B in Arabidopsis cold acclimation were published [45,46,47]. Under cold stress, the B-phosphorylated blue light receptor *CRY2* is stabilized and in this state inhibits the degradation of *HY5*. I Intact *HY5* positively regulates freezing tolerance by modulating the expression of a set of *COR* genes, including some active in anthocyanin biosynthesis [48]. This process is mainly independent of the *CBF* pathway [46,47]. To date, all we know about the role of B in the cold hardening process of cereals is that B up-regulates the *COR14b* protein (one of the targets of *CBF*s) in barley under cold conditions [49] and *CBF14* in wheat and barley even at higher temperatures, which are not suitable for inducing cold acclimation (20 and 15 °C) [6]. It was not revealed in these papers whether or not B is also able to induce freezing tolerance, similarly to FR.

These published results suggested that B supplementation coupled with the previously applied FR enrichment could affect the regulation of the cold acclimation process in barley, at least in the early pre-hardening phase. The impact of FR enrichment at the hormone and lipid levels and the expression of the key hormone/lipid metabolism-related genes have already been elucidated in winter barley leaves at moderate (15 °C) and low (5 °C) temperatures [3,5]. Thus, as a sequel, in this study we investigated whether an extra B supplementation would be able to further improve the well-documented freezing tolerance enhancing effect of FR-enriched W in barley plants. To explore the effect of the modified light environment on the pre-hardening process in detail, total hormone and lipidome analyses were conducted.

## 2. Results

### 2.1. Determination of Frost Tolerance

**Figure 1:**
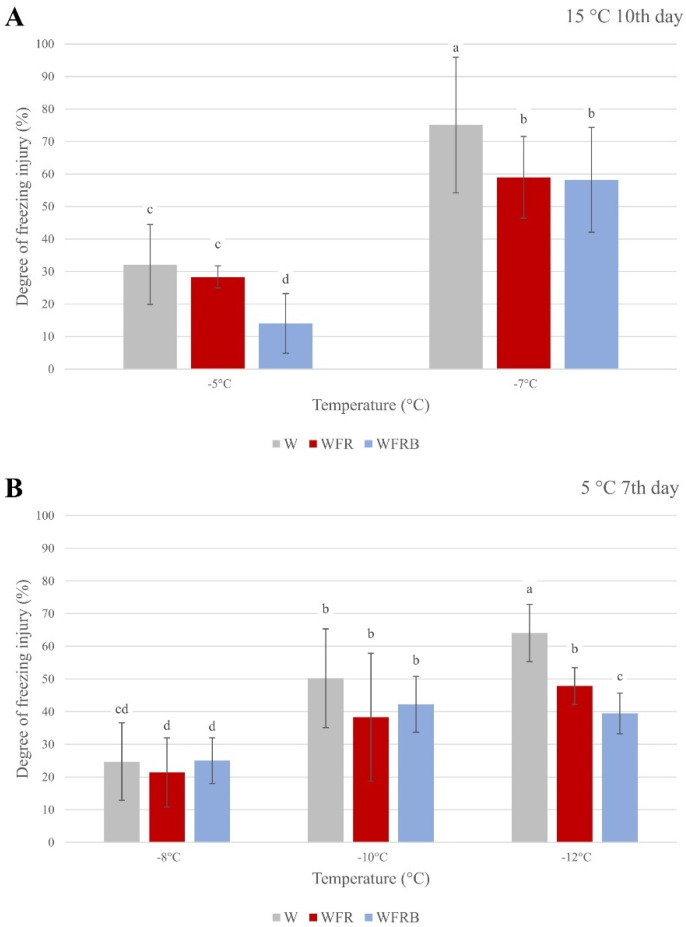
**The effects of the supplementary far-red and blue light treatments on freezing tolerance under various temperatures.** The X−axis shows the freezing temperatures, whereas the Y−axis refers to the relative conductance values (percentage of lethality). The plantlets were grown under a 12 h photoperiod. W: white light, WFR: far-red-enriched white light, WFRB: far-red- and blue-enriched white light. In each case, the samples were collected on the last day of the treatments. (**A**) 10th day at 15 °C temperature; (**B**) 7th day at 5 °C. The data and error bars, which represent the standard deviation, originated from three biological replicates. Statistical analysis was performed with Kruskal−Wallis test. Values indicated with different letters are significantly different from each other at *p* ≤ 0.05 levels.

The frost tolerance levels of the barley plants were tested by evaluating the degree of freezing injury (Figure 1). These results showed clear differences between the light treatments. In the case of the plants grown at 15 °C, FR treatment resulted in significantly decreased conductivity of the leaf samples compared to those grown in control W after freezing at −7 °C. If additional blue light was applied, it further increased frost tolerance in the case of plants grown at 15 °C. Growing barley plants for a week at 5 °C (which can be considered as pre-hardening) increased the frost resistance of the plants regardless of the light treatments used. The effect of the modulated light became visible after freezing at −10 and −12 °C. At both temperatures, the low R:FR ratio was very effective according to the relative conductance values, but the addition of B light even significantly further increased the frost tolerance at −12 °C.

### 2.2. Hormones

**Figure 2:**
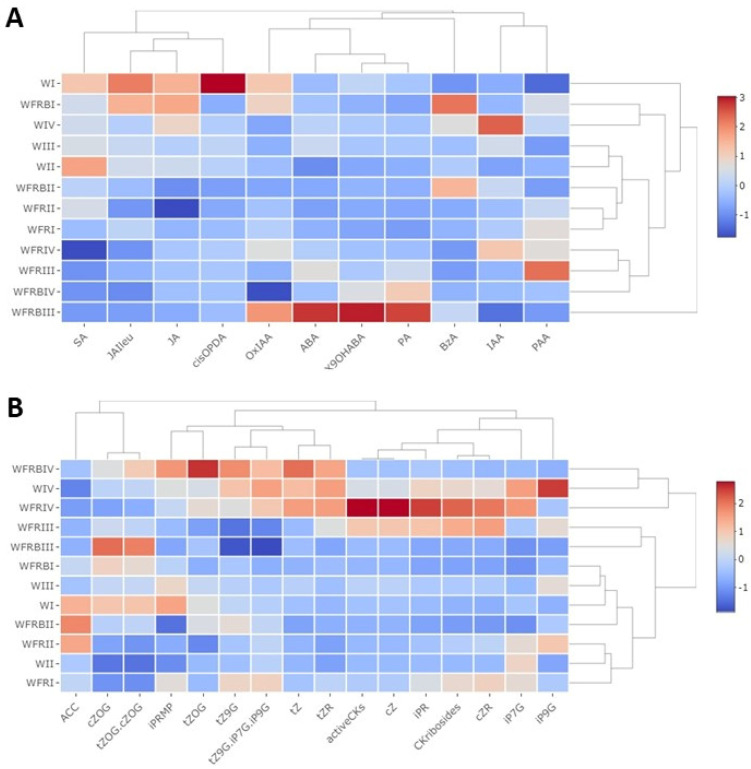
**The effect of supplementary FR and Blue light at normal and low temperature on hormone concentrations.** Leaf samples were collected six to eight hours after the start of illumination. The plantlets were grown under a 12 h photoperiod. (**A**) fraction A, eluted with methanol containing ABA, IAA, SA, and JA; (**B**) fraction B, eluted with 0.35 M NH4OH in 60% methanol containing CKs. W: white light, WFR: far-red-enriched white light, WFRB: far-red and blue-enriched white light. I−1 day at 15 °C, II−10 days at 15 °C, III−1 day at 5 °C, IV−15 days at 5 °C. The data originated from three to five biological replicates. The values on the X and Y axis outside of the heat map refer to the distance or proximity of data after hierarchical clustering.

The supplementation of WFR with B had a strong positive effect on the levels of ABA, the key hormone in the responses to abiotic stresses. Transient ABA elevation was observed after 1 day at 5 °C (Figure 2A). It was considerably stronger than in the case of mere FR supplementation. Massive stimulation of ABA biosynthesis by WFRB at low temperature is indicated also by a high elevation of ABA catabolites, phaseic acid (PA) and ABA hydroxyderivative (9OH-ABA). Significantly enhanced levels of both metabolites were found even after a 7-day treatment. Interestingly, SA levels were slightly decreased by low temperature under W, but were further decreased by FR and FRB supplementation. The amount of one of the SA precursors, benzoic acid (BzA) was significantly affected (increased) only by WFRB at 15 °C. At 15 °C, accumulation of JA as well as that of its active conjugate jasmonate-isoleucine (JA-Ile) were decreased by FR supplementation. In the case of WFRB, the effect was observed after 7 days. At 5 °C, JA and JA-Ile contents were decreased under all light treatments, but to a much stronger extent under WFR and WFRB. The accumulation of the JA precursor cisOPDA was downregulated at 15 °C in WFR and WFRB during the whole experiment, at 5 °C in all variants. The levels of ethylene precursor 1-aminocyclopropane-1-carboxylic acid (ACC) were transiently suppressed by WFR and WFRB supplementation at 15 °C. At 5 °C, ACC was decreased in all light conditions.

The auxin indole-3-acetic acid (IAA) was not affected by light spectra at 15 °C. At low temperature, a negative effect of WFR and especially of WFRB was detected (Figure 2A). This corresponds well to the increase of IAA metabolite Ox-IAA under WFRB (after 1 day) and WFR (after 7 days). In contrast to IAA, weak auxin phenylacetic acid (PAA) was upregulated by WFR after 1 day at both temperatures.

The amount of the physiologically most active CK, trans-zeatin (tZ) was elevated at 5 °C during cold acclimation (after 7 days) under all light treatments (the most under WFRB). The levels of other active CKs, isopentenyladenine (iP) and especially cis-zeatin (cZ) were moderately enhanced at 5 °C in W, increasing over time (Figure 2B). The effect was much stronger at WFR. In contrast, WFRB was associated with their suppression. Decrease of cZ under WFRB was associated with elevation of its inactive metabolite O-glucoside (cZOG). CK ribosides followed the same trend as the corresponding bases; the promotive effect of FR was, however, also observed at 15 °C (in the case of iPR and cZ).

### 2.3. Lipid Results

#### 2.3.1. Mass Spectroscopic Analysis of Isolated Lipids

The lipid composition of the barley leaves was examined by mass spectroscopy. We compared the quantitative and qualitative lipid composition of barley leaves grown at 5 and 15 °C and under three different light illuminations (W, WFR, WFRB).

**Figure 3:**
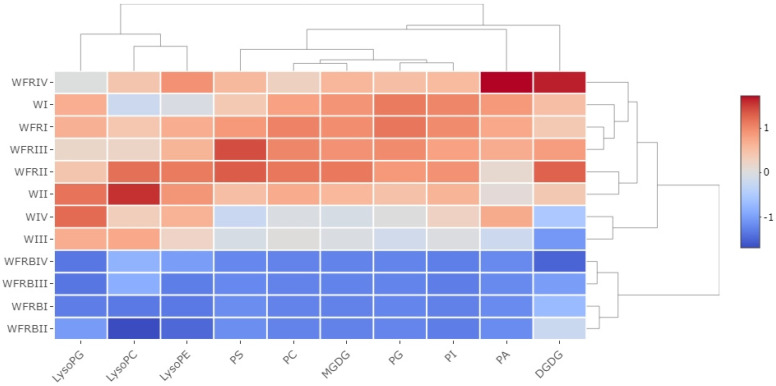
**Lipid class distribution in barley after supplementary FR and Blue light illumination with cold treatments.** The samples were illuminated with white light, white light supplemented with far-red light (WFR or combined far-red with additional blue light (WFRB). In addition to light treatment, two different temperatures were used (5 and 15 °C). The samples were taken on the first and last days of the treatments, which was ten days in the case of 15 °C and seven in the case of 5 °C. In all cases, the leaf samples were cut from the central part of the second youngest leaf.

Based on the results of the PCAs, we observed clear differences between the individual light and temperature treatments. The results showed that in terms of light alterations, temperature change and length of the treatments, the strongest effect was caused by light modifications. This phenomenon was especially strong in the case of WFRB treatments regardless of the temperature change or treatment duration. WFRB light changed the total lipid content negatively. Strong regulation was also caused by the WFR treatment, which alone inflicted positive influence on many lipid classes. Although the low temperature and the length of the light treatments did not affect the lipid content to such an extent, it can be stated that they had a modifying effect in both cases (Supplementary Figure S2). This phenomenon was further confirmed by the heat map covering the changes in lipid classes (Figure 3).

All experimental variants differing in temperature or sampling time in relation to the WFRB treatment are clearly classified into a distinct cluster. There was always a decreasing trend in the measured lipid classes compared to W. In the case of WFR treatment the exact opposite effect was found. Lysophosphatidyl lipids, which are primarily precursors of PC and PE [14], were clustered in a well-differentiated group in terms of individual lipid classes. A significant decrease in the total LPC content could be observed not only in WFRB light but also after FR supplementation, which mainly occurred after the temperature drop. According to the total LPE content, the amount of lipids changed significantly after ten days, primarily in the case of WFR and WFRB treatments. We can see a significant decrease in the WFR treatment at 5 °C, which was strengthened by WFRB light.

#### 2.3.2. Changes in the Composition of Lipid Classes

**Figure 4:**
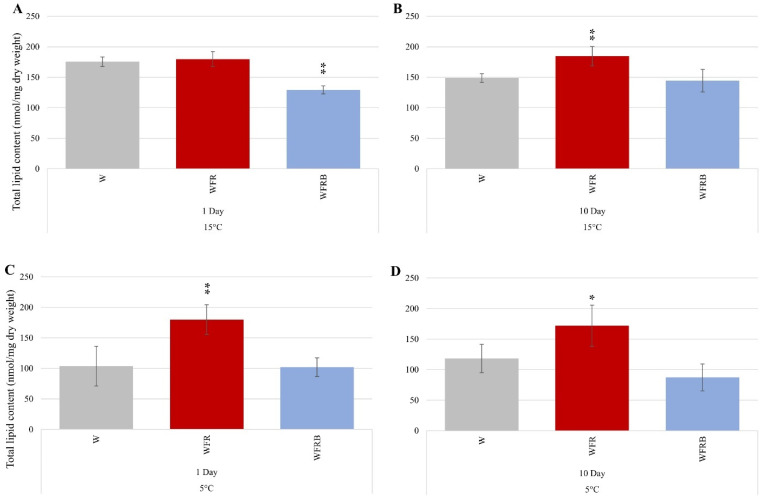
**Changes in total lipid content after supplementary FR and Blue light illumination under cold treatments.** The signal was expressed as detected signal/mg dry weight (Y axis). The samples were illuminated with white light, white light supplemented with far-red light (WFR) and combined far-red with additional blue light (WFRB). In addition to light treatment, two different temperatures were used (5 and 15 °C). The samples were taken on the first and last days of the treatments, which was ten days in the case of 15 °C and seven in the case of 5 °C. (**A**) 1st day at 15 °C; (**B**) 10th day at 15 °C; (**C**) 1st day at 5 °C; (**D**) 7th day at 5 °C. In all cases, the leaf samples were cut from the central part of the third leaf. Statistical analysis was performed with multiple-way ANOVA and Dunnett’s post-hoc test using white light (W) samples as controls. Measurements were made on 3 to 5 independent biological replicates. Significance levels are indicated by * *p* < 0.1 and ** *p* < 0.05.

Figure 4 shows the total lipid content determined by relating the signal value to dry weight. It could be seen that light quality had a remarkable effect on total lipid content. As a result of WFR illumination, the total lipid content increased, both on the tenth day of the 15 °C treatment, and on the first and seventh days at 5 °C. Under WFRB illumination, the total lipid content decreased regardless of the temperature.

Since the total lipid content changed because of the light treatments, we also calculated the quantitative changes of the individual lipid families (Figure 3B). The total amount of MGDG did not change in response to FR supplementation. However, if B was added to the light mix, then the amount of MGDG increased. The total amount of DGDG did not change significantly in response to the treatments.

Among the less abundant lipid families, PG content decreased under B supplementation compared to the other light treatments at 15 °C in the first day. In contrast, on the first day after the temperature drop (5 °C), its quantity elevated due to the addition of FR and also FRB.

In the case of PC, after one day each enriched light mixture resulted in an enormous elevation, independent of temperature. It should be noted that this increase was maintained longer at 5 °C under WFRB light, whereas it completely disappeared under WFR. At 15 °C, B supplementation reduced the amount of PE. In addition to the cold treatment, PE decreased under all the illuminations.

PA changed in a way opposite to that expected. The amount of PA decreased with FR supplementation, and this decrease was more profound under added B illumination at every temperature. Interestingly, the total amount of PS did not change during the treatments.

Since the DGDG and MGDG are the major lipid components of the chloroplast double-layered membrane, we calculated their ratio in each treatment. The DGDG/MGDG ratio showed a reduction compared to the control W light (Table 1). Comparing the individual treatments, this decrease was the most significant after addition of B.

We also examined the sum of PC and PE (Table 1). FR supplementation decreased their values under longer cold treatment. The WFRB treatment positively affected this content at all temperature conditions.

The ratio of PC to PE serves as an index to determine the degree of membrane degradation by frost injury. Although the PC/PE ratio was not changed by the WFR treatment, WFRB illumination doubled it at any tested time and temperature (Table 1).

#### 2.3.3. The Distribution of Lipid Species within the Different Lipid Classes

During treatments, the 34:4 subspecies within the MGDG family (Table S1) gave a constant low signal. The amount of 36:6, 36:5 and 36:4 was decreased by WFRB light independently from the temperature applied. The amount of the 34:1 subspecies was enormously increased by WFRB.

Supplementary Table S1 shows the effect of the treatments on the composition of the DGDG lipid family. A significant decrease was observed for the 34:3, 34:2, 34:1, 36:4 and 36:3 species at low temperature. The group including 36:6 and 36:2 was increased both under low R:FR and WFRB, regardless of temperature. At 5 °C, the amount of 38:6 lipids decreased under illumination with added FR, whereas it increased in WFRB.

The results of the PE lipid family can be seen in Supplementary Table S1. The amount of the 34:3 and 36:6 subspecies decreased at 15 °C, whereas the amount of 36:3 increased. The 34:2 subspecies was positively influenced by WFR and WFRB at both temperatures. The amount of the 36:4 group increased during the WFRB treatment at 5 °C but decreased due to WFR treatment at 15 °C in the long run.

The composition of the PC lipid family showed the greatest changes after WFRB light, since the 36:6, 36:5, 36:4 and 34:2 subspecies were increased as a result of this treatment, whereas the amount of 34:3 decreased especially under low temperature.

#### 2.3.4. Changes in the Unsaturation Level of Fatty Acids

In order to determine the lipid unsaturation levels of the cell membranes, the double bond index (DBI) parameter was used, and the total double bond values of the examined lipids were determined. We did not observe any significant deviation in total DBI due to temperature changes under W, but light treatments caused significant differences. Table 2 shows the total DBI content. The amount of DBI was significantly increased by WFRB independently of the temperature. Interestingly, WFR treatment resulted in DBIs very similar to the values obtained under control conditions at 15 °C.

Further analysis of the double bond index of the lipids studied showed that these alterations were mainly caused by changes occurring in the MGDG, PC and PE lipid families (Table 2).

In MGDG, DBIs were changed significantly only under WFRB, which resulted in an elevation that was retained even after the cold treatment. The PC family exhibited a very similar trend. In addition to that increase, very slight changes also occurred due to WFR, but only at 15 °C. In the PE family DBIs exhibited an opposite tendency. As a result of WFRB, a large reduction was detected under almost all conditions. Nonetheless, FR supplementation slightly increased the DBI at 15 °C and 5 °C after one day of treatment.

## 3. Discussion

It has been already established that the initiation of the cold acclimation process is regulated not only by temperature, but also by photoperiod, light intensity, and light quality [10,50,51]. The extent of leaf damage by a stress may be estimated by electrolyte flow out of the cells and thereby by increased conductivity of the distilled water in which the leaf segments float [52,53]. This method was applied in our previous studies to verify the effect of the FR light addition, which increased frost resistance [8,9]. However, very little information is available on the effect of B on frost resistance, especially in cereals.

In *Arabidopsis thaliana*, B was found to positively influence plant readiness to cold acclimation, especially in the early stages of cold response [47]. B reportedly increased frost resistance based on Fv/Fm values, even after one day of treatment, but did not reduce electrolyte efflux based on conductivity measurements. Our data on winter barley clearly indicates that the frost resistance level is higher based on the reduction of electrolyte leakage at low temperature under WFRB light. This can be explained by the fact that the increased ratio of B light in the incident W spectrum significantly affected both phytohormone and lipid metabolism. These changes probably promoted plant readiness to respond to cold stress and increased the fluidity of the chloroplast and cell membranes.

Due to their sessile lifestyle, plants need to respond quickly to any change of various environmental factors. In our previous study, in accordance with other publications, we demonstrated that the additional FR light positively influenced the freezing tolerance of plants by significant modulation of many phytohormones [5,10,54]. Since the phytohormone profiles imposed by FR light in this study are very similar to our previously described data, we will primarily discuss the effects of supplementary B to W enriched with FR light (WFRB).

In *Arabidopsis thaliana*, it is becoming more and more certain that monochromatic B has a positive effect on frost tolerance [47,55]. B was reported to positively affect cold-stress related proteins, i.e., those associated with defense in *Arabidopsis thaliana* [46]. In accordance with this finding, the cold-induced increase in ABA content in barley leaves was further increased by B, as the ABA level was considerably higher after WFRB than in the case of mere WFR supplementation. Moreover, in barley seeds, exposure to B upregulated the expression of the *HvNCED1* gene in the embryo, resulting in an increase in the embryonic ABA level [12,56].

In contrast to ABA, salicylic acid (SA) levels were slightly decreased by low temperature under W illumination, and a further decrease was observed after application of WFR and WFRB light. JA, as well as its active conjugate JA-Ile showed a very similar tendency. It seems that strong stimulation of the ABA signaling pathway may have a negative effect on rival hormone pathways associated with biotic stress responses. Suppression of SA and JA levels by FR light under low temperature may resemble the shade avoidance syndrome. Suppression of JA- and SA-dependent defenses during shade response was found to be associated with enhanced plant vulnerability to herbivore and pathogen attack at low R:FR ratio [35,57]. This correlates with what we reported in our previous article, i.e., that the shade avoidance response was prioritized over plant protective responses in the case of SA and JA [5].

At normal temperature, IAA was not affected by any light modification, but a negative effect of WFR and especially of WFRB was found at 5 °C (Figure 2A). This is rather interesting, since IAA is generally elevated during the shade avoidance response caused by FR. One of the possible reasons for this alteration may be the high light intensity used (W supplementation with FR), which represents a large difference relative to the process of shade avoidance [45,58]. Moreover, the low temperature resulting in suppressed plant growth may have a negative effect on IAA levels. ABA-deficient rice mutants had reduced IAA content, which led to increased cold resistance [34]. When comparing IAA dynamics, it seems that at low temperatures plants prioritize defence mechanisms over shade avoidance [9,35].

It has been reported that the *HY5* transcription factor is responsible for the convergence between CKs and blue light signals during photomorphogenesis [59]. An upregulation of *HY5* in Arabidopsis leaves correlated with an increase in CK contents [59]. However, the shade avoidance response is associated with stabilization of PIFs and destabilization of *HY5* as well as with enhanced CK degradation by cytokinin oxidase/dehydrogenase [40]. Under cold stress, B was reported to inhibit *HY5* degradation. At low temperature *HY5* can also activate ABA synthesis by up-regulating the expression of *SlNCED6* as well as ABA signaling by directly binding to the promoter of *ABI5* [60]. As the barley *HY5* gene has not been characterized, it is difficult to evaluate *HY5* abundance under WFRB conditions. It seems that, similarly to the regulation of auxin content, low temperature and relatively high light intensity affect the CK pool differently from the shade avoidance response. It is evident that the combination of low temperature and FR light represents an important signal to overwintering plants in temperate zones to start cold acclimation. WFR was associated with high up-regulation of less active, stress-related cis-zeatin, its riboside and iPR. WFRB resulted in quite a different pattern. After one day, cis-zeatin metabolites (especially cZOG) were accumulated under WFRB. Later, enhanced levels of cis-zeatin and its riboside were not observed under B supplementation to WFR. This is in accordance with a recent analysis of the expression of CK-related genes like CRF and type-B ARR transcription factors, which are predominantly down-regulated under blue light [46,61]. However, the positive effect of WFRB on frost tolerance seems to be reflected by faster cold acclimation associated with elevated content of the highly physiologically active tZ and its reversible metabolite tZOG (in comparison with W and WFR). Thus, in all light variants prolonged cold treatment resulted in at least partial acclimation accompanied by (moderate) CK increase.

It has already been observed that changes in MGDG and DGDG content resulted in enhanced drought and frost tolerance in *Arabidopsis thaliana* [62,63]. On the contrary, testing of different chromosome introgression lines from a cross of monocots (Lolium multiflorum × Festuca arundinacea) gave a result opposite to that observed in the case of drought tolerance [13]. These observations and our results on barley support the hypothesis that the mechanism of stress adaptation of monocots differs from the dicot model mentioned. Therefore, it is very likely that the increase in MGDG concentration in the chloroplast membrane induced by FR and especially by B supplementation may have contributed to the increase in frost tolerance of barley leaves.

Changes in PC and PE content seem to be much more important from the point of view of membrane integrity. It is known that the total amount of PC and PE in rye and wheat leaves increases during cold acclimation [64,65,66]. In our system, regardless of temperature, the amount of PC increased significantly due to additional B, whereas FR alone did not affect its accumulation. PC is known to play a significant role in the formation of the double membrane and in maintaining its integrity [67]. Therefore, it is likely that an increase in PC due to B supplementation under low R:FR ratio contributes significantly to an increase in frost tolerance. Similarly, the PE content was not significantly affected by WFR treatment. On the contrary, its quantity decreased as a result of WFRB treatments. Since PE is a phospholipid that does not form a bilayer double membrane with PC [67], this change may also contribute to increasing frost tolerance.

Recently more and more evidence has accumulated about the importance of PA as a new lipid class involved in many cellular functions in plants. Moreover, PA participates in regulatory signaling pathways in cell growth and biotic and abiotic stress responses as well [68]. It is well established that PA is formed with the help of phospholipases and/or lipid kinases. Many studies also mentioned PA as a key signaling molecule during plant salt stress responses [69,70]. PA also acts as a marker in frost tolerance studies, since higher levels of PA are associated with a poor outcome for plants after frost injury [14]. During cold exposure, the PA level was also much higher in Eutrema salsugineum [71]. In our experiments, the PA level did not change as a result of WFR, but it decreased due to WFRB. This trend was independent of temperature. It is important to point out that the reduced PA content under the influence of higher blue light ratio may indicate better readiness to cold acclimation. These results may indicate that the elevated B supplementation could strongly regulate a key element of the signal transduction pathway, thereby changing the viability of plant grown under high blue illumination. The amount of PA, similar to several lipid families, changed significantly as a result of the treatments, which may be related to the fact that it is an important precursor in many lipid biosynthesis pathways.

MGDG is a non-bilayer-forming (NBL) lipid, whereas DGDG is a bilayer-forming (BL) lipid. A decrease in the ratio of these lipids was observed in plants grown at low temperatures [14,72]. During cold treatments, a drop in the MGDG/DGDG ratio and a change in PG levels are generally detectable, but may differ depending on the plant species [71,73,74]. According to our experimental results, the DGDG/MGDG ratio (Table 1) decreased upon every light treatment but had no negative effect on the membrane stability of barley leaves. WFRB illumination reduced this ratio more strongly than WFR treatment. This reduction probably contributed significantly to the increased photosynthetic efficiency observed as compared to the control W or the WFR treatment.

It is worth mentioning that during our experiment the plants reached only a certain pre-hardened state, since 3 to 5 weeks of cold treatment would be necessary to develop complete cold acclimatization [66]. The PC/PE ratio did not change significantly in response to FR treatment; the additional B in the mix significantly reduced the amount of PE, but enhanced the PC content, thereby decreasing their ratio (Table 1). In contrast to PE, PC belongs to the group of bilayer-forming lipid classes, and it is the most abundant membrane lipid in monocot plants [65]. It is likely that increasing the PC/PE ratio is one of the main reasons why B supplementation effectively increases the frost tolerance of barley plants [75], because the higher is this ratio, the less damaged is the membrane [76].

In MGDG, based on the distribution of the length and the unsaturation level of the carbon chains, we can consider the reduction of 36:6, 36:4 and 36:5 groups as a response to light treatments at 15 °C, whereas 34:1 and 34:3 groups are probably changed by the combination of light and low temperature. For DGDG, the 36:6 and 36:2 groups showed a temperature-independent increase, whereas 38:6 responded oppositely to added FR illumination and WFRB light which was discussed above. These results suggest that under B-enriched illumination, the saturation level of thylakoid membrane lipids increased, but this did not occur universally. Among the fatty acid chains of the PC lipid family, 36:6 increases in all cases, whereas 36:4 was decreased at 15 °C but raised at 5 °C under WFR illumination, in the contrast to WFRB. However, the exact function of these subfamilies is currently unknown.

Our results indicate that different light and temperature treatments caused changes mainly in the most abundant lipid subspecies of the lipid families. Due to these changes, the structure and fluidity of not only the chloroplast but also the thylakoid membrane were changed. The unsaturation levels of the different lipid families can also be determined from the data available on their distribution. Therefore, we can identify the typical changes that take place in the distribution of the number of double bonds in different fatty acid chains.

It has been repeatedly confirmed that the unsaturation of the acyl chains connected to the head group of fatty acids plays a decisive role in the development of cold acclimation by modulating membrane fluidity [77]. DBI can be used to describe this phenomenon, since a higher value indicates a more fluid membrane structure. Based on the total DBI, we can say that WFR treatment with an increased R:FR ratio did not affect this parameter, whereas WFRB did. DBI increased during the application of B in the light mix.

A detailed analysis revealed that the values of MGDG, PE and PC significantly elevated due to the increase in the proportion of B in the incident light. These lipid families are involved in the regulation of membrane dynamics along with DGDG [78]. According to our previous results, the higher DBI of membrane-forming lipids enhanced the tolerance to low temperature-induced photoinhibition in transformed cyanobacteria [79]. We could see a similar increase in the lipid content of the tested barley leaves during the application of different light regimes. It was previously described that the increment in the unsaturation level of PE is related to cold acclimation in Arabidopsis and cereals as well [64,80,81,82]. In barley, a frost tolerant variety had a much higher unsaturated free fatty acid content than a frost-sensitive one [83]. It was suggested that cold-induced changes in PE family subspecies can be used as a diagnostic marker for predicting frost tolerance in cereals [66]. Our results strengthen this statement. We assume that the decreased ratio of R:FR in WFR changed the PE subspecies and led to an increased cold acclimation level of the frost-tolerant barley already at 15 °C, which can be further enhanced with added B (WFRB) at 5 °C.

## 4. Materials and Methods

### 4.1. Plant Materials and Growth Conditions

Italian winter habit barley seedlings with good cold tolerance, *Hordeum vulgare* spp. *vulgare var*. Nure were used. After the germination (3 days), 600 seedlings were planted into wooden boxes (30 cm × 25 cm × 10 cm) and filled with soil from the field. The boxes were placed into a PGV-36 growth chamber (Conviron PGV36; Controlled Environments Ltd.; Winnipeg, MB, Canada) equipped with a modular LED light ceiling. During this period, plants were grown at constant 15 °C, with 12-h photoperiods for fourteen days at 250 µmol m^−2^ s^−1^ (PPFD) light intensity. For this developmental stage, the light was provided by a continuous wide-spectrum LED (Philips Lumileds, LXZ25790-y) which was considered as our control W light. The plants were irrigated with 50% Hoagland medium 3 times a week.

### 4.2. Light and Temperature Conditions during Experimental Treatments

After the two-week developmental phase, half of the plants remained at 15 °C, whereas the other half were moved to 5 °C. At both temperatures, plants were separated into three areas. In the first area the same W was used at 250 µmol m^−2^ s^−1^ intensity, whereas in the second zone W was enriched with FR illumination (WFR) with a narrow-band 750 nm LED (Edison Edixeon, 2ER101FX00000001), which modified the R:FR ratio to 0.5. The R:FR ratio was calculated according to Smith [84]. In the third zone W intensity was lowered, and W was supplemented not only with FR but also with B (WFRB) with a very narrow 410 nm (Philips Lumileds, LXZ1—PR01) monochromatic LED to achieve the same light intensity (R:FR ratio remained to 0.5). The spectral composition of the used light regimes can be found in Supplementary Material (Figure S1). The light treatments were 10 days long at 15 °C and 7 days long at 5 °C.

Samples for hormone, lipid and gene expression analysis were collected during the first and the last days of each treatment between 6 and 8 a.m. Samples for relative conductance measurements were collected only on the last day of each treatment.

### 4.3. Determination of Frost Tolerance in Leaf Samples

Freezing tests were performed according to [52]. For the evaluation of freezing tolerance, about two-mm-long leaf segments were excised from each treatment. In all the cases altogether twelve leaf segments were added into 14-mL Falcon tubes (Thermo Fisher Scientific Inc., Wilmington, MA, USA) from four different plants in five biological repetitions per treatment for each freezing temperature. The freezing program was implemented in a GP200-R4 liquid freezing system (Grant Instruments, Shepreth, UK) as described in our previous works [5,9], with final selected temperatures of −5 and −7 °C for 15 °C samples and −8, −10, and −12 °C for 5 °C samples. Then, electrolyte leakage measurements were performed by a conductometer (Mikro KKT, Budapest, Hungary) [5,9]. For data analysis, Multi-Sample Conductometer version 1.0 (Intron Software, Biological Research Centre, Szeged, Hungary (Copyright© L. Menczel, 2002) was used. The degree of freezing injury was calculated according to the formula of Prášil and Zámečník [85].

### 4.4. Hormone Analysis

For the hormone analysis the leaf samples were purified and analyzed according to Dobrev et al. and Dobrev and Vankova [86,87]. Hormones were purified and homogenized from 100 mg leaf tissue samples with a ball mill (MM301, Retsch) and extracted in cold (−20 °C) methanol/water/formic acid (15/4/1 *v*/*v*/*v*). The following labelled internal standards (10 pmol/sample) were added: ^13^C_6_-IAA, ^2^H_2_-OxIAA (Cambridge Isotope Laboratories); ^2^H_4_-SA (Sigma-Aldrich, St. Louis, MI, USA); ^2^H_3_-PA (phaseic acid), ^2^H_3_-DPA (dihydrophaseic acid), ^2^H_4_-7OH-ABA, ^2^H_5_-ABA-GE (ABA-glucosyl ester) (NRC-PBI), ^2^H_6_-ABA, ^2^H_5_-JA, ^2^H_5_-transZ, ^2^H_5_-transZR, ^2^H_5_-transZ7G, ^2^H_5_-transZ9G, ^2^H_5_-transZOG, ^2^H_5_-transZROG, ^2^H_5_-transZRMP, ^2^H_3_-DHZ, ^2^H_3_-DHZR, ^2^H_3_-DZRMP, ^2^H_7_-DZOG, ^2^H_3_-DHZ9G, ^2^H_7_-DZOG, ^2^H_6_-iP, ^2^H_6_-iPR, ^2^H_6_-iP7G, ^2^H_6_-iP9G, ^2^H_6_-iPRMP (Olchemim). After that the extracts were purified using a mixed mode reverse phase–cation exchange SPE column (Oasis-MCX, Waters). Two hormone fractions were sequentially eluted.

(1) fraction A, eluted with methanol containing ABA, IAA, SA, and JA.
(2) fraction B, eluted with 0.35 M NH4OH in 60% methanol containing CKs.

The hormone metabolites were analyzed using HPLC (Ultimate 3000, Dionex) coupled to a hybrid triple quadrupole/linear ion trap mass spectrometer (3200 Q TRAP, (Applied Biosystems, Foster City, CA, USA). Quantification of hormones was done using the isotope dilution method with multilevel calibration curves (R2 > 0.99). Data processing was carried out with Analyst 1.5 software (Applied Biosystems). Data are presented as means ± standard error. In all instances five biological replicates were analyzed.

### 4.5. Lipid Isolation

For lipid extraction, barley leaves were collected in liquid nitrogen separately from five different plants, with 0.2 g fresh weight. Total lipid extracts were obtained from frozen samples (stored at –80 °C) as described in Kovács et al. (2020) [3]. Mass spectrometric analysis of all lipid extracts was performed using an MS-based method and carried out by the analytical laboratory of the Kansas Lipidomics Research Center (https://www.k-state.edu/lipid/analytical_laboratory/lipid_profiling/index.html). The raw results were accessed on 5 February 2020.

### 4.6. ESI-MS/MS Lipid Profiling

An automated electrospray ionization—tandem mass spectrometry procedure was used. Data collection and analysis besides acyl group identification were performed as described by Kovács et al. (2020) [3]. Lipid extracts were continuously injected into the ESI source on a triple quadrupole MS/MS (API4000, AB Sciex, Framingham, MA, USA). Data processing is described on the website of the Kansas Lipidomics Research Center (https://www.k-state.edu/lipid/analytical_laboratory/lipid_profiling/index.html). The processing method were accessed on 9 October 2020.

### 4.7. Double Bond Index (DBI)

The DBs of all individual lipid molecules were calculated as the amount of lipid molecules and the average number of double bonds/acyl chain, where the average number of double bonds/acyl chain was calculated by dividing the number of double bonds in lipids by the number of acyl chains in molecular species. The DB indexes of all the lipid classes were calculated so that each lipid molecular species in the given class was the sum according to the following equation [Σ (normalized signal intensity %/lipid species dry weight × number of double bonds)]/100 [3].

### 4.8. Statistical Analysis

The lipid, hormone and freezing test data were examined with various statistical methods to reveal the potential significant effects of the different treatments. Where assumptions of ANOVA were not met, Kruskal-Wallis tests were carried out to determine if there are any significant differences between the different temperature and light treatments in the case of both lipid, hormone, and freezing test data. The effects of light and temperature treatments were checked with both PCA analysis and Heatmap analysis. Statistical analyses were performed in an R statistical computing environment using the following packages: “ggplot2”, “factoextra”, “agricolae” and “heatmaply”.

## 5. Conclusions

According to our best knowledge, these are the first results in cereals to show that the combined effects of cold and light modification (especially with supplementary blue light) can greatly influence the phytohormone pools and lipid contents, which eventually lead to better readiness to avoid frost injury. In accordance with this statement, the cold-induced increase in ABA content in barley leaves was further increased by B light, as the ABA level was considerably higher after WFRB than in the case of mere FR supplementation. By comparing IAA dynamics, it seems that at low temperatures plants prioritize defence mechanisms over shade avoidance. In addition, WFRB light also positively affected the physiologically active tZ and its reversible metabolite tZOG, which can be associated with faster cold acclimation. The hormonal results clearly show that the increased frost tolerance under combined blue and far-red light supplementation can occur because blue light represses the shadow avoidance syndrome induced by FR. During cold acclimation of plants, the composition of lipid membranes changes dynamically. Based on our results of the conductance test we can say that blue light supplementation promotes the preservation of the integrity of the chloroplast and thylakoid membranes, since lipid metabolism was highly influenced by the addition of blue to the W+FR mix. WFRB illumination reduced the DGDG/MGDG ratio more strongly than WFR treatment. This reduction probably contributed significantly to the better photosynthesis efficiency under cold stress conditions. The PC/PE ratio did not change significantly in response to FR treatment, but was significantly increased by the additional B. It is likely that this enhancement is one of the main reasons why B supplementation effectively increases frost tolerance. PA content was significantly decreased only by B treatment, which demonstrates the integrity of membrane structure. A detailed analysis revealed that the DBI values of MGDG, PE and PC were significantly elevated only due to the increased proportion of B in the incident light. Similarly, to the modulation of plant membranes under prolonged acclimation to cold, this alteration also brings about increased membrane fluidity.

## Figures and Tables

**Table 1:** **Changes in the DGDG/MGDG and PC/PE ratios in barley after supplementary FR and Blue light treatments at different temperatures.** The signals were expressed as a percentage of the total lipid content detected (total lipid signal percentage (%)). In all cases, the treatments were compared to the first-day samples in the control W-lighted zones (15 °C 1 D). The samples were illuminated with white light, white light supplemented with far-red light (WFR) or combined far-red with additional blue light (WFRB). In addition to light treatment, 5 °C treatments were also made. The samples were taken on the first and last days of the treatment, which was ten days at 15 °C and seven days at 5 °C. In all cases, the leaf samples were cut from the central part of the second youngest leaf. Statistical analysis was performed with multiple-way ANOVA followed by Dunnett’s post-hoc test. Measurements were made on 3 to 5 independent biological replicates (±SE). Significance levels are indicated by * *p* < 0.1 and ** *p* < 0.05 and *** *p* < 0.01.

		W	WFR	WFRB
DGDG/MGDG ratio	15 °C 1 D	0.39 ± 0.00	0.37 ± 0.01	0.33 *** ± 0.01
	15 °C 10 D	0.46 ± 0.01	0.41 *** ± 0.01	0.31 *** ± 0.00
	5 °C 1 D	0.49 ± 0.01	0.42 * ± 0.01	0.44 ** ± 0.03
	5 °C 7 D	0.58 ± 0.01	0.56 ± 0.00	0.49 ± 0.01
PC/PE ratio	15 °C 1 D	1.3 ± 0.1	1.2 ± 0.0	2.3 ** ± 0.1
	15 °C 10 D	1.3 ± 0.0	1.2 ± 0.0	3.7 *** ± 0.5
	5 °C 1 D	1.2 ± 0.0	1.1 ± 0.0	2.4 *** ± 0.0
	5 °C 7 D	1.0 ± 0.0	0.9 ± 0.0	1.9 *** ± 0.1
PC + PE	15 °C 1 D	17.2 ± 0.7	19.1 ± 0.3	20.2 * ± 0.4
	15 °C 10 D	18.7 ± 1.3	19.5 ± 1.3	15.8 ± 1.0
	5 °C 1 D	19.4 ± 1.0	19.6 ± 0.0	20.8 ± 0.5
	5 °C 7 D	17.4 ± 0.3	15.1 ± 1.2	24.0 *** ± 1.1

**Table 2:** **Changes of MGDG, PE and PC lipid classes and total DBI content in barley leaves after supplementary FR and Blue light treatments at different temperatures.** In all cases, the treatments were compared to the first-day samples in the control W (15 °C 1 D). The samples were illuminated with white light, white light supplemented with far-red light (WFR) or combined far-red with additional blue light (WFRB). In addition to light treatment, 5 °C treatments were also made. The samples were taken on the first and last days of the treatment, which was ten days at 15 °C and seven days at 5 °C. In all cases, the leaf samples were cut from the central part of the second youngest leaf. Statistical analysis was performed with multiple-way ANOVA followed by Dunnett’s post-hoc test. Measurements were made on 3 to 5 independent biological replicates (±SE). Significance levels are indicated by * *p* < 0.1 and ** *p* < 0.05 and *** *p* < 0.01.

Lipid Family	Treatment	W	WFR	WFRB
Total	15 °C 1 D	4.81 ± 0.24	4.77 ± 0.24	5.03 *** ± 0.29
	15 °C 10 D	4.82 ± 0.24	4.90 ± 0.25	5.13 *** ± 0.31
	5 °C 1 D	4.80 ± 0.24	4.77 ± 0.24	4.91 ± 0.27
	5 °C 7 D	4.63 ± 0.21	4.72 ± 0.22	4.84 * ± 0.25
MGDG	15 °C 1 D	2.40 ± 0.05	2.39 ± 0.04	3.00 *** ± 0.05
	15 °C10 D	2.40 ± 0.03	2.53 ± 0.11	3.22 *** ± 0.05
	5 °C 1 D	2.30 ± 0.06	2.33 ± 0.07	2.64 ** ± 0.10
	5 °C 7 D	1.95 ± 0.08	2.04 ± 0.15	2.44 *** ± 0.06
PC	15 °C 1 D	0.33 ± 0.03	0.36 ± 0.01	0.50 ** ± 0.01
	15 °C 10 D	0.37 ± 0.03	0.39 ± 0.03	0.43 ± 0.01
	5 °C 1 D	0.38 ± 0.02	0.36 ± 0.04	0.52 *** ± 0.02
	5 °C 7 D	0.30 ± 0.01	0.26 ± 0.03	0.58 *** ± 0.01
PE	15 °C 1 D	0.28 ± 0.01	0.32 ± 0.00	0.23 ± 0.02
	15 °C 10 D	0.29 ± 0.02	0.32 ± 0.02	0.14 *** ± 0.03
	5 °C 1 D	0.32 ± 0.02	0.34 ± 0.03	0.22 *** ± 0.01
	5 °C 7 D	0.31 ± 0.01	0.28 ± 0.03	0.30 ± 0.03

## Data Availability

Not applicable.

## Supplementary Materials

The following supporting information can be downloaded at: https://www.mdpi.com/article/10.3390/plants12010040/s1.
