# Supplementary material for: The Effect of White Light Spectrum Modifications by Excess of Blue Light on the Frost Tolerance, Lipid- and Hormone Composition of Barley in the Early Pre-Hardening Phase
Source: Plants (Basel). 2022 Dec 22;12(1):40. doi: 10.3390/plants12010040 (PMC9823678; doi:10.3390/plants12010040)
Supplement: Supplementary file 1 [file plants-12-00040-s001.zip › Supplementary Materials.pdf]

Supplementary Materials

The effect of white light spectrum modifications by excess of blue light on the frost tolerance, lipid- and hormone composition of barley in the early pre-hardening phase

Mohamed Ahres, Tamás Pálmai, Teréz Kovács, László Kovács, Jozef Lacek, Radomíra Vanková, Gábor Galiba, Péter Borbély

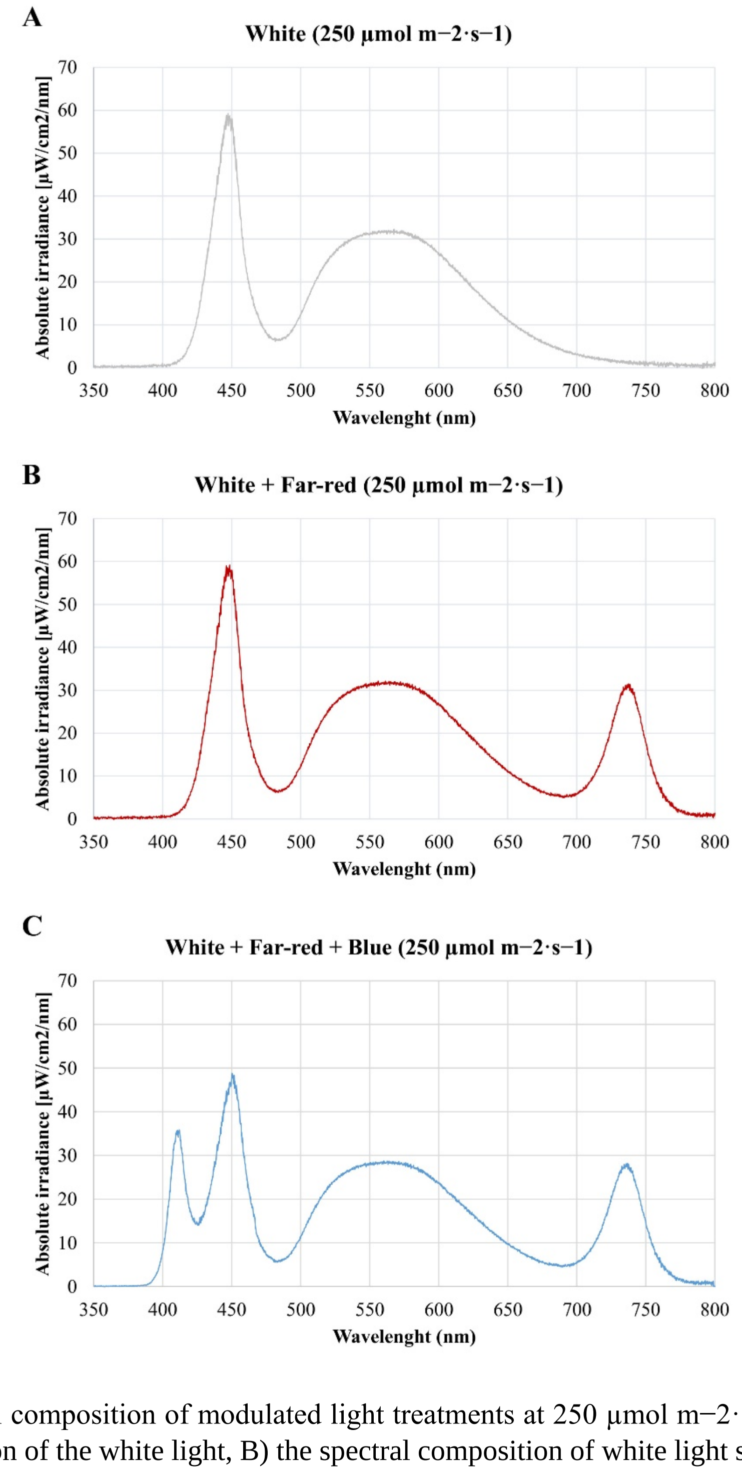

Figure S1: Spectral composition of modulated light treatments at 250 µmol m−2·s−1 intensity. A) the spectral composition of the white light, B) the spectral composition of white light supplemented by far-red light, C) the spectral composition of white light supplemented with far-red and monochromatic blue (410nm) light.

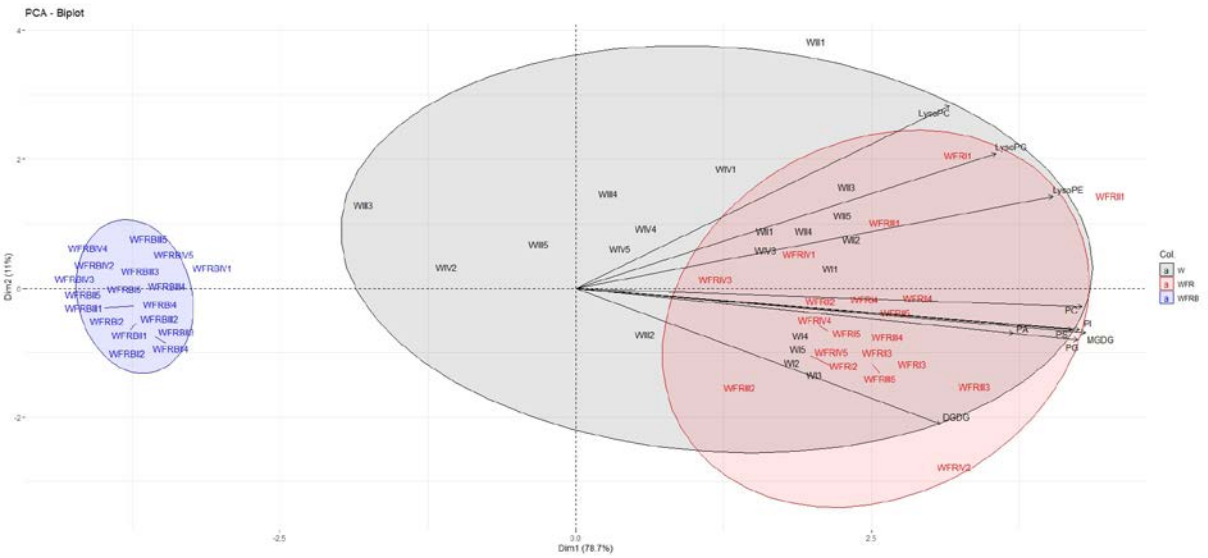

Figure S2: Principal component analysis on lipid classes under different light compositions. Grey circle: white light, Red circle: far-red-enriched white light, Blue circle: far-red and blue enriched white light. The statistical analyses were performed in R statistical computing environment using the following packages: “ggplot2”, “factoextra” and “agricolae”.
