# Supplementary figures and images for: The Effect of White Light Spectrum Modifications by Excess of Blue Light on the Frost Tolerance, Lipid- and Hormone Composition of Barley in the Early Pre-Hardening Phase
Source: Plants (Basel). 2022 Dec 22;12(1):40. doi: 10.3390/plants12010040 (PMC9823678; doi:10.3390/plants12010040)

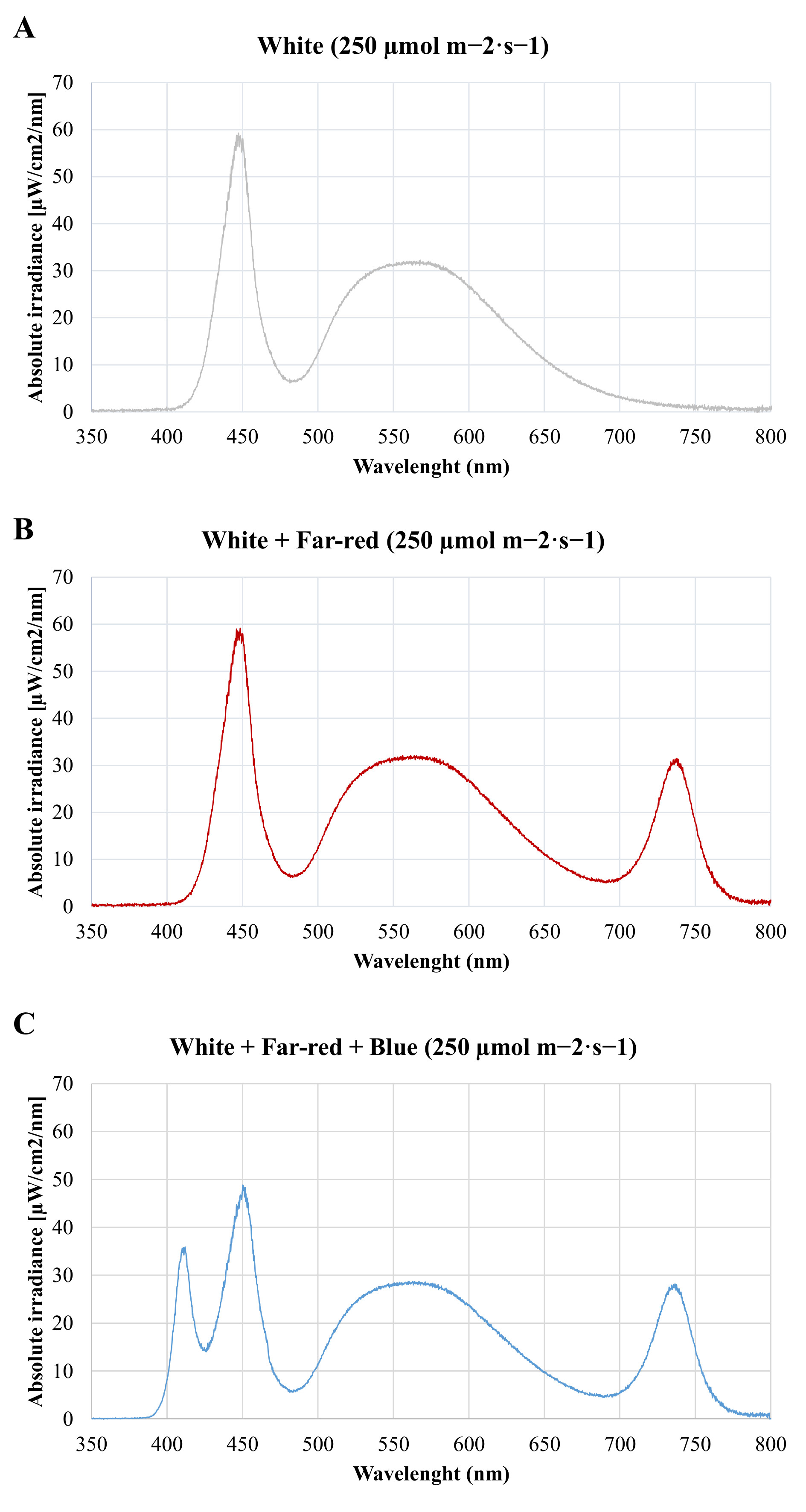

Supplement: Supplementary file 1 [file plants-12-00040-s001.zip › Figure S1.jpg]

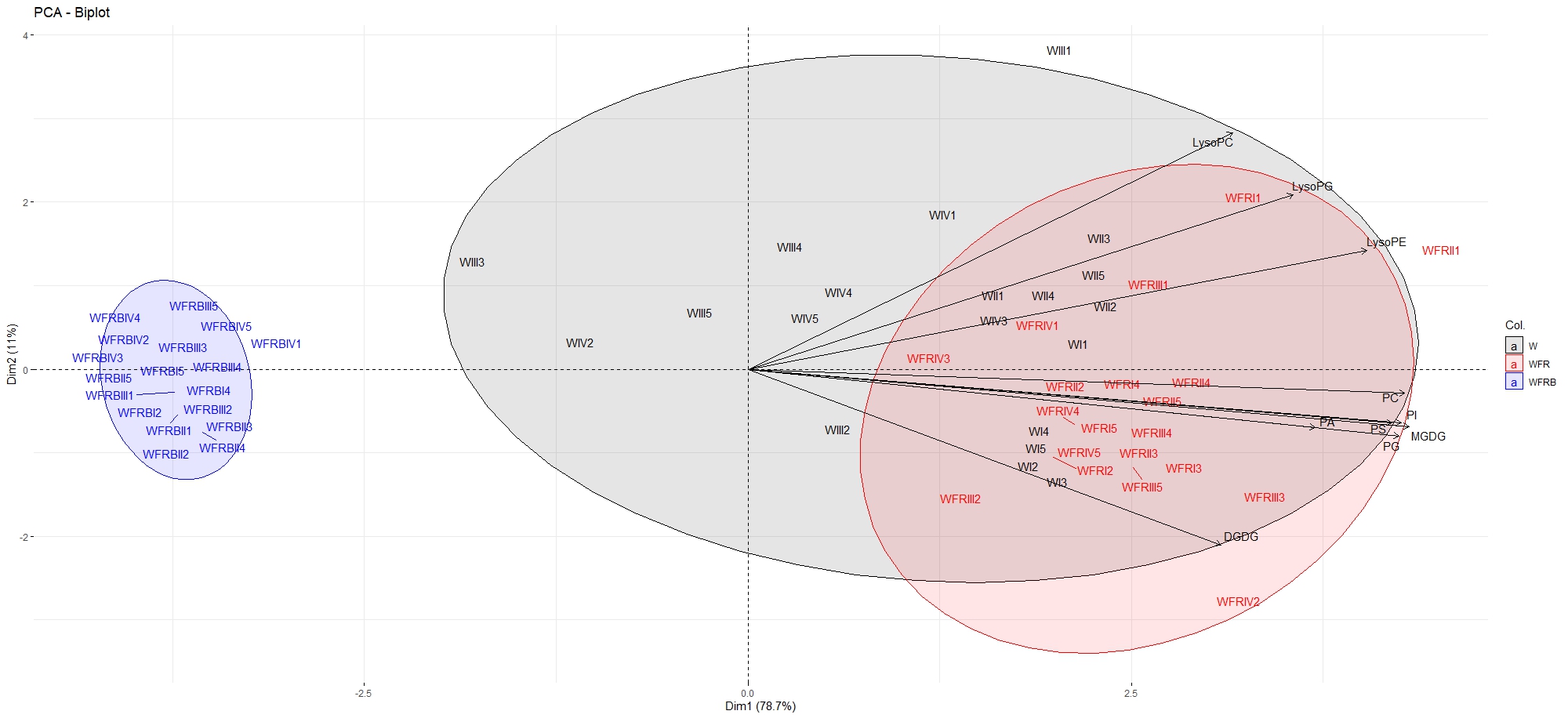

Supplement: Supplementary file 1 [file plants-12-00040-s001.zip › Figure S2.jpeg]
